# Towards the automation of early-stage human embryo development detection

**DOI:** 10.1186/s12938-019-0738-y

**Published:** 2019-12-12

**Authors:** Vidas Raudonis, Agne Paulauskaite-Taraseviciene, Kristina Sutiene, Domas Jonaitis

**Affiliations:** 1 Department of Automation, Kaunas University of Technology, 51367 Kaunas, Lithuania; 2 Department of Applied Informatics, Kaunas University of Technology, 51368 Kaunas, Lithuania; 3 Department of Mathematical Modelling, Kaunas University of Technology, 51368 Kaunas, Lithuania

**Keywords:** Deep learning, Location detection, Embryo development, Image recognition, Multi-class prediction

## Abstract

**Background:**

Infertility and subfertility affect a significant proportion of humanity. Assisted reproductive technology has been proven capable of alleviating infertility issues. In vitro fertilisation is one such option whose success is highly dependent on the selection of a high-quality embryo for transfer. This is typically done manually by analysing embryos under a microscope. However, evidence has shown that the success rate of manual selection remains low. The use of new incubators with integrated time-lapse imaging system is providing new possibilities for embryo assessment. As such, we address this problem by proposing an approach based on deep learning for automated embryo quality evaluation through the analysis of time-lapse images. Automatic embryo detection is complicated by the topological changes of a tracked object. Moreover, the algorithm should process a large number of image files of different qualities in a reasonable amount of time.

**Methods:**

We propose an automated approach to detect human embryo development stages during incubation and to highlight embryos with abnormal behaviour by focusing on five different stages. This method encompasses two major steps. First, the location of an embryo in the image is detected by employing a Haar feature-based cascade classifier and leveraging the radiating lines. Then, a multi-class prediction model is developed to identify a total cell number in the embryo using the technique of deep learning.

**Results:**

The experimental results demonstrate that the proposed method achieves an accuracy of at least 90% in the detection of embryo location. The implemented deep learning approach to identify the early stages of embryo development resulted in an overall accuracy of over 92% using the selected architectures of convolutional neural networks. The most problematic stage was the 3-cell stage, presumably due to its short duration during development.

**Conclusion:**

This research contributes to the field by proposing a model to automate the monitoring of early-stage human embryo development. Unlike in other imaging fields, only a few published attempts have involved leveraging deep learning in this field. Therefore, the approach presented in this study could be used in the creation of novel algorithms integrated into the assisted reproductive technology used by embryologists.

## Background

Infertility is a growing problem worldwide. According to the World Health Organization, one in every six couples has issues leading to infertility problems. It has been noted that the global in vitro fertilisation (IVF) market is expected to grow at an approximated 10% compound annual growth rate between 2018 and 2026 [1]. Geographically, Europe dominates the market by capturing the largest share, which is driven by low fertility rates, government financial support for the adoption of IVF and other fertility treatments, and the increasing success rate of IVF methods. According to the forecasts [2], the Asia-Pacific region is anticipated to demonstrate rapid growth in the foreseeable future. Causes of infertility are numerous, potentially including factors such as anatomical or genetic problems, physiological dysfunction, sexually transmitted diseases, endocrinological or immunological problems, and many more. Moreover, the rising trend towards delaying pregnancy due to career concerns, financial reasons or not finding the right partner has also increased the need for IVF services. The success of IVF procedures is closely linked to many biological and technical issues. The fertilisation and in vitro culturing of embryos are dependent upon an environment that should be stable and correct with respect to temperature, air quality, light, media pH and osmolality. After fertilisation, an embryo that develops normally will continue to divide, growing to the blastocyst stage by the fifth or sixth day; however, only one-third of all embryos are capable of reaching this stage [3]. The success rate of IVF procedures resulting in a pregnancy varies between age group in average it is less than 52% [4]. For this reason, more than one embryo is transferred, which subsequently increases the risk of multiple pregnancies. In fact, more than 30% of IVF-induced pregnancies are multiple-infant births. For this reason, embryo viability is monitored by an embryologist during the IVF procedure. Nevertheless, embryo assessment is subjective and based on limited observations if it is performed visually by placing the fertilised embryo under a microscope once to a few times per day.

**Fig. 1:**
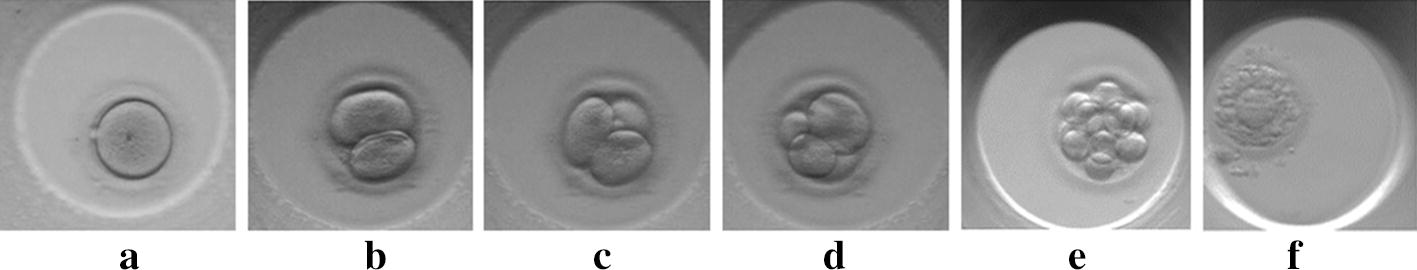
Images of embryo development stages: **a** 1-cell embryo; **b** 2-cell embryo; **c** 3-cell embryo; **d** 4-cell embryo; **e** > 4-cell embryo; **f** no visible cells

Time-lapse (TL) systems developed over recent years (with or without computer algorithms) provide a massive number of digital images of embryos at frequent time intervals, thus enabling embryologists to assess the quality of the embryos without physically removing them from their culture environment [5]. Embryos can be transferred to the uterus at the cleavage stage (Day 2 or 3, Fig. 1b–e) or blastocyst stage (Day 5, Fig. 1f). Transferring embryos at the blastocyst stage may increase the likelihood of selectively transferring viable and genetically normal embryos [6]. The correct identification of cell number creates presumptions for determining the timing parameters from time-lapse imaging, such as the duration between different stages, which was approved as being significant in the evaluation of embryo quality [7].

Despite all of the recent advances in computer vision research, the automatic detection and tracking of cells remain challenging. This task is complicated by the topological changes of tracked objects (cell division) in addition to the possible presence of randomly appearing noise in the images. In comparison, many other medical imaging applications exist, where the variability of relevant data, such as target object, surrounding structures or image acquisition parameters, have a large impact on the decisions made by domain experts. For example, a previous experiment [8] emphasised the need to study longitudinal retinal nerve fibre layer (RNFL) thickness changes in patients with open-angle glaucoma, while the need to develop a single software package to automatically determine differences in aortic diameter from multiple scans of the same patient was presented recently [9]. Moreover, the algorithm to be developed should process a large number of image data files of different quality in a reasonable amount of time. Unlike in other fields of image recognition, far too little attention has been paid to the use of artificial intelligence in the detection of human embryo quality development.

Deep learning is now a state-of-the-art artificial intelligence model across a variety of domains and is seen as a key technique for future human-support technologies. As indicated by previous studies [10, 11], deep learning methods—more specifically convolutional neural networks (CNNs)—hold huge potential for medical imaging technology, medical diagnostics and healthcare in general. Unlike conventional machine-learning techniques, deep neural networks simplify the feature engineering process, provide abstract learning through a hierarchical representation of the data, efficiently deal with vast amounts of data and demonstrate their superiority in detecting abnormalities in medical images. Recently, an approach named STORK was developed that can be used for unbiased and automated embryo assessment using TL images [12]. They formulated a binary classification problem focusing on good- and poor-quality embryo assessment, which was tackled using deep neural networks, more specifically Inception-V1 architecture. In their research, authors used a large collection of human embryo time-lapse images (approximately 50,000 images) from a high-volume fertility centre in the US. The authors highlighted that STORK was able to predict blastocyst quality with an area under curve (AUC) of $>0.98$, which is a very promising result. In the same manner, Iwata et al. [13] examined the use of deep learning on images of human embryos for predicting good- and poor-quality embryos. They also referred to other studies [14–16] that utilised artificial intelligence approaches for quality prediction or grade classification with varying degrees of success. Comparatively, in another study [17], the authors used a list of the main morphological features of a blastocyst with the aim of automating embryo grading using support vector machine (SVM) classifiers. They reported accuracies ranging from 0.67 to 0.92 for embryo development classification. Overall, these studies represent attempts to develop reliable algorithms for the prediction of a two-class problem.

Notably, the application of artificial intelligence focusing on multi-class prediction remains scarce. The recent study proposed a standalone framework based on Inception-V3 CNNs as the core to classify individual TL images up to the 4-cell stage for mouse and human embryos, respectively [18]. In their work, 31,120 images of 100 mouse embryos and 661,060 images of 11,898 human embryos cultured in the TL monitoring system were analysed. The experimental study on the test set demonstrated an average classification accuracy of 90% when the model was applied to predict individual images up to the 4-cell stage, while accuracy of 82% was achieved when it was applied to identify embryos up to the 8-cell stage. In this context, a three-level four-class embryo stage classification method based on the Adaboost ensemble was proposed with the aim to identify the number of cells at every time point of a TL microscopy video, which resulted in an average accuracy of 87.92% for human embryos, but exhibited only 20.86% accuracy for 3-cell detection [19]. To the best of our knowledge, these are the few known works that have addressed the identification of early-stage embryo development by formulating a multi-class prediction problem.

In line with these findings, the present study contributes to this field by proposing a model to automate the monitoring of early-stage human embryo development by focusing on the prediction of the cell number during the division process for up to 5 days. This involves segmenting embryos from the image and then predicting defined number classes that relate to the embryo development stages (i.e. 1-cell, 2-cell, 3-cell, 4-cell and > 4-cell; see Fig. 1) using CNNs. Whereas one of the key elements of the system is the detection of embryo location in an image, the algorithm is proposed for this purpose. It first determines the rough embryo location using a Haar feature-based cascade classifier and then specifies its accurate location by means of the radiating lines. The use of this algorithm allowed us to achieve an accuracy of over 92% in predicting the early stages of embryo development.

## Results

Images of early-stage embryo development were captured using a ESCO Miri TL incubator system with an integrated camera, which has a 2.35-megapixel image sensor that provides a 1936 × 1216 pixels (px) resolution output (2.48 px = 1.00 $\upmu$m). It captures the embryo image in 7 different focal planes. The camera is capable of capturing 47 frames per second. However, recording of the development process is performed at 5-min intervals since embryo development is a relatively slow process. The experiment included 300 TL embryo development sequences for a total of 114,793 frames (18.73%, 25.45%, 9.35%, 20.65% and 25.82% of the data set for 1 to > 4-cell stages, respectively).

**Fig. 2:**
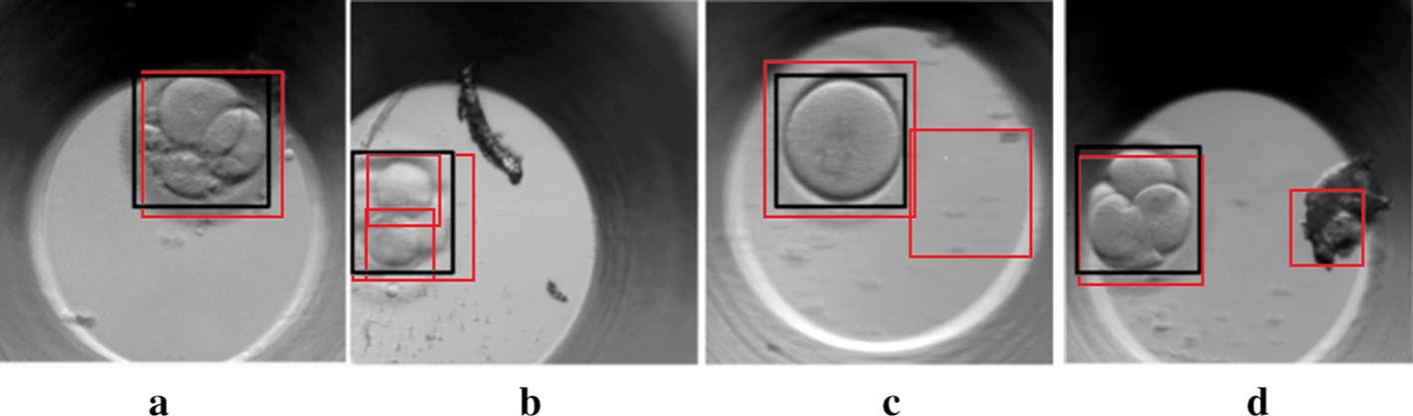
Illustration of accurate localisation (black rectangle) and unsuccessful localisation (red rectangle) of early-stage embryo including **a** a detection of wrong area; **b** a determination of individual cells; **c** a determination of empty areas; **d** a detection of not relevant objects

**Fig. 3:**
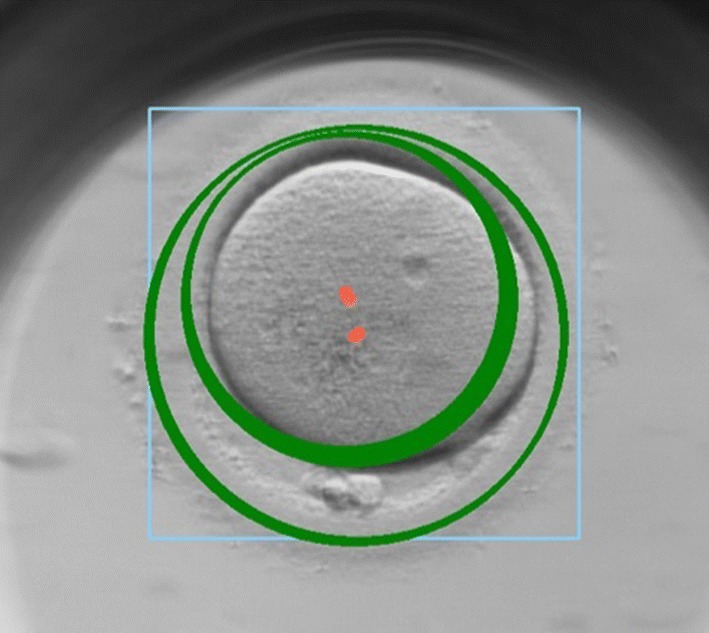
Case of well detected embryo location: the green circle should fit the boundaries of the embryo; the red point illustrates the centre of detected region

First, the automatic detection of embryo location in the image was performed using the cascade classifier. It was noted that mostly linear diagonal Haar-like features were leveraged by the algorithm (see Fig. 2a). Unfortunately, the location of the entire embryo was not always successfully detected, as illustrated in Fig. 2. For instance, (a) a wrong area of the entire embryo is determined; (b) the individual cells are detected but not the entire embryo; (c) the empty areas are determined; or (d) the objects of no interest are also detected. Therefore, the algorithm developed by the authors was used for embryo location detection. The proposed embryo location detection algorithm was considered successful for a problem if the entire embryo and its fused membrane were correctly identified in the image. The thickness of the membrane, its brightness and the number of granules are among the top criteria for assessing the quality of an embryo. That is why their detection is a crucial step in the present research. In Fig. 3, a well-localised embryo is highlighted by a green circle.

The algorithm proposed here includes the drawing of radiating lines, which are used to visualise the gradient direction, in which the gradient values are computed from the pixel values. Higher gradient values are obtained when the line is radiating over the embryo boundaries, where digital images have discontinuities. This allows us to indicate the physical limits (a boundary) of embryo.

**Fig. 4:**
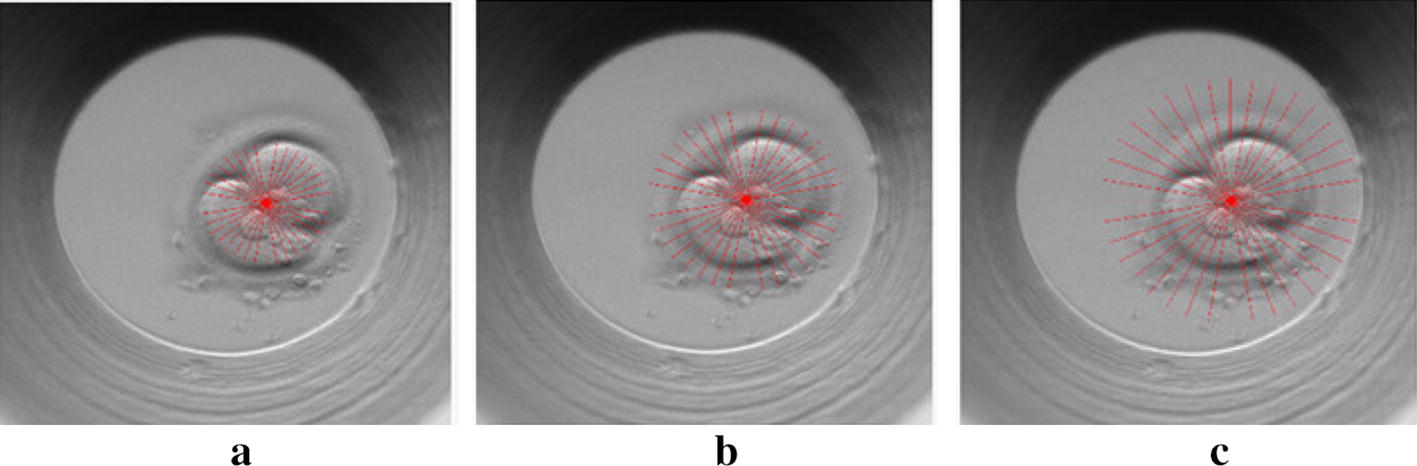
Radiating lines with lengths of 40 $\upmu$m (**a**), 60 $\upmu$m (**b**), and 80 $\upmu$m (**c**)

The length of the line and the angle between radiating lines are the main parameters to be considered. The change of line length affects the area of the image to be covered, while the change of angle between lines determines a different density to be explored in the image. Figure 4 demonstrates the scattering of lines in the image for different lengths of radiating lines, given in $\upmu$m.

**Fig. 5:**
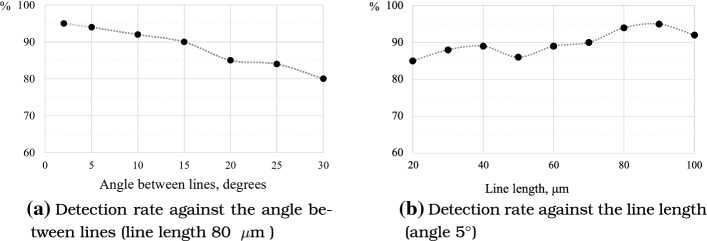
Investigation of automatic detection of embryo location

The ability of the proposed algorithm to correctly detect an entire embryo location is demonstrated in Fig. 5, where different radiating line lengths and the angle between them are investigated.

As illustrated in Fig. 5, the correct location detection rate for the entire embryo is rather high. However, the algorithm is more sensitive to changes in angle size between lines (see Fig. 5a). The increase of angle negatively impacts the detection quality. On the other hand, the number of points to be processed increases rapidly if the angle is decreased. Figure 5b shows that the detection rate is above 90% if the line length is over 70  $\upmu$m when the angle is 5$^{\circ }$. Typically, an embryo covers an area from 101 × 101 $\upmu$m up to 121 × 121 $\upmu$m.

Next, the classification of embryo development stages is explored. In the present research, five classes were specified in order to represent each early stage of embryo development (i.e. 1-cell, 2-cell, 3-cell, 4-cell, > 4-cell). The obtained confusion matrix for two CNNs architectures, such as AlexNet and VGG16, is presented in Table 1.

**Table 1:** Confusion matrices: each column shows the reference, while numbers running diagonally show the percentage of correct classification for every class considered in the experimental study

Prediction		Reference				
		1	2	3	4	> 4
VGG architecture						
1		0.930	0	0	0	0
2		0.023	0.943	0.036	0.019	0.027
3		0.022	0.029	0.932	0.022	0.027
4		0.025	0.027	0.032	0.957	0.028
> 4		0	0	0	0.001	0.917
AlexNet architecture						
1		0.910	0	0	0	0
2		0.030	0.944	0.039	0.030	0.026
3		0.029	0.029	0.920	0.032	0.026
4		0.031	0.027	0.041	0.937	0.031
> 4		0	0	0	0.001	0.917

It can be seen that the classification performance is generally quite high. The comparison of two classifiers was performed by computing the confusion matrix-based performance measures [20, 21]. All experiments demonstrated in the paper have been performed forming training and testing image data sets in a ratio of 70:30, respectively. The tenfold cross-validation has been performed in order to estimate the prediction accuracy of a classifier using CNNs. The stratified version of this method was selected so that the correct proportion of each of the class values would be assigned to each fold. The results of stratified cross-validation are provided in Appendix C, where Table 4 reports the classification accuracy averaged over all tenfold achieved using the selected CNN architectures. One can see that VGG model achieved average accuracy of 0.936 and its standard deviation of 1.2%. Comparatively, AlexNet model resulted in average classification accuracy of 0.927 and smaller standard deviation of 0.8%. Unsurprisingly, the 3-cell stage was the most challenging since the lowest average accuracy accompanied with the largest deviation was achieved when either model was used. On the whole, cross-validation results give us assurance that the accuracy estimate is stable.

Table 2 highlights that the overall performance in terms of selected measures using the AlexNet architecture is slightly worse when compared to results from using the VGG architecture. It is evident that no difference exists between micro-accuracy and macro-accuracy. Compared to a macro-F1 score, micro-F1 obtains larger values for both CNNs architectures used in the experiment. Since F1 score is a balance between precision and recall, Table 3 was created to reveal the classifier performance by class to address these measures.

**Table 2:** Overall performance

	VGG	AlexNet
Macro-accuracy	0.936	0.927
Micro-accuracy	0.935	0.925
Macro-F1	0.926	0.919
Micro-F1	0.963	0.952

**Table 3:** Class-specific performance

VGG			AlexNet	
	Precision	Recall	Precision	Recall
1	1.000	0.930	1.000	0.910
2	0.927	0.943	0.915	0.944
3	0.790	0.932	0.767	0.920
4	0.901	0.957	0.888	0.937
5	0.999	0.917	0.999	0.917

Table 3 shows that precision is rather low for the third class, which defines the embryo stage as having three cells. Since micro-averaging favours classes with a larger number of instances, the final estimate was influenced by good performance for the classification of the other classes.

The training and testing data sets consist of images of different embryos (more than one patient). The quality of images is different, because of several reasons, such as the image is out of focus, the embryo is partly occluded with foreign objects, the embryo is captured outside of the image sensor, etc. The image data set was carefully examined and labelled by a skilled embryologist. Poor data such as low-resolution images, images without an embryo or images with an occluded embryo with a material that does not belong to the embryo were excluded. The duration of the 3-cell stage is approximately 8–10 times shorter than, for example, the 2-cell stage; as such, the number of samples of the 3-cell stage in the image data set is smaller. Therefore, the number of samples at other cell stages (1-cell, 2-cell, 4-cell, or higher) was limited to the number of 3-cell samples.

## Discussion

The evaluation of early-stage embryo quality has been a matter of debate for many years. Using novel computer vision algorithms, various techniques have been developed to maximise the effectiveness of assisted reproductive technology. The use of TL imaging might increase the IVF success rate since this new approach allows the detection of abnormal behaviour in developing embryos.

TL imaging enhanced the selection criteria of the transferable embryo since the development of the embryos is observed to be more accurate. The quality of an embryo can be described by the KIDScore grading method [22]. It demonstrates that the embryo transition or cleavage from one stage to another has a certain optimal time. If an embryo cleaves from one cell to more cells too quickly or too slowly, then the embryo has a low probability for transfer. The authors of this paper aim to evaluate the embryo development with the use of deep learning techniques in order to automate the assessment of embryo quality at early development stages. The proposed method consists of two major steps: the embryo localisation into 2D image space and embryo stage classification.

The accurate localisation of the embryo into 2D image is very important task. It is done using the combination of Haar-like features and computation of the gradients on cell boundaries. Haar-like features are sensitive to the contrast of the image. These features provide more accurate output when captured embryo image has sharp edges. Lower accuracy is acquired, when image is out of focus and embryo boundaries are fuzzy. The appearance of foreign objects in the cultivating dish is not common thing. However, when foreign objects appear they can partly occlude an embryo or be next to it. Foreign objects can be mistaken as an embryo or can distort final classification result by occluding the embryo. The authors of the research work are proposing to use as many as possible Haar-like feature to lower risk of the false classification. More Haar-like features describe more embryo-specific characteristics in the image and it becomes separable from foreign object. Notably, the proposed approach has certain limitations. A deep learning-based method is only as smart and accurate as the data provided in training. For this research, the model was trained using TL images from a private IVF clinic. The training database used to construct a decision-making core could be expanded by capturing more possible variations of different embryos. Synthetic images of human embryo cells could be generated using Generative Adversarial Networks (GANs) due to a lack of real-world data [23], however the highest results of 96.2 % have been achieved for 1-cell embryo images only. Specifically, unrealistic synthetic images consisting of more cells could be created using GANs algorithm. For example, evaluating 4-cell images, 80 % accuracy was obtained (i.e. one out of five images was generated inaccurately). GANs are very suitable for expanding the variability of the training database where all variations of objects are allowed [24]. The method has shown its superiority in generating data for medical imaging in solving unsupervised classification problem, which suffers from a small training set and includes only two classes of images (i.e. cancer or not cancer) [25]. However, our research goal is to find embryos with the best quality among others for human IVF while solving a multi-class prediction problem, therefore learning using only realistic images is reasonable. Therefore, it might be interesting to explore different algorithms for generating partial or hybrid data set, where original and synthetic data are used in learning. This could be the next step towards being able to build a fully automatic monitoring system for evaluating embryo quality.

## Conclusion

The present study has reported the problems and suggested methods to automate early-stage human embryo detection. The proposed algorithm consists of two components, namely embryo localisation in the image and classification of embryo development stage. The detection of embryo location has been successful by using the improved object detection algorithm. First, the rough centre of the embryo is identified using Haar-like features. Then, a more accurate location of the embryo is computed by leveraging the radiating lines. The experimental investigation showed that detection accuracy of at least 90% was reached using radiating lines of length 80 $\upmu$m placed at every 5°. It was also determined that 80 $\upmu$m is the optimal line length (radius detected from the rough centre of an embryo), which is sufficient to wrap the entire embryo in the image. Embryo stage classification performance had an overall accuracy above 92%, which was achieved for both CNN architectures considered in the paper. The most problematic was the third class, which defines the 3-cell stage. This might have been caused by this stage usually being short compared to the other classes defined in the paper.

## Methods

### Time-lapse system

**Fig. 6:**
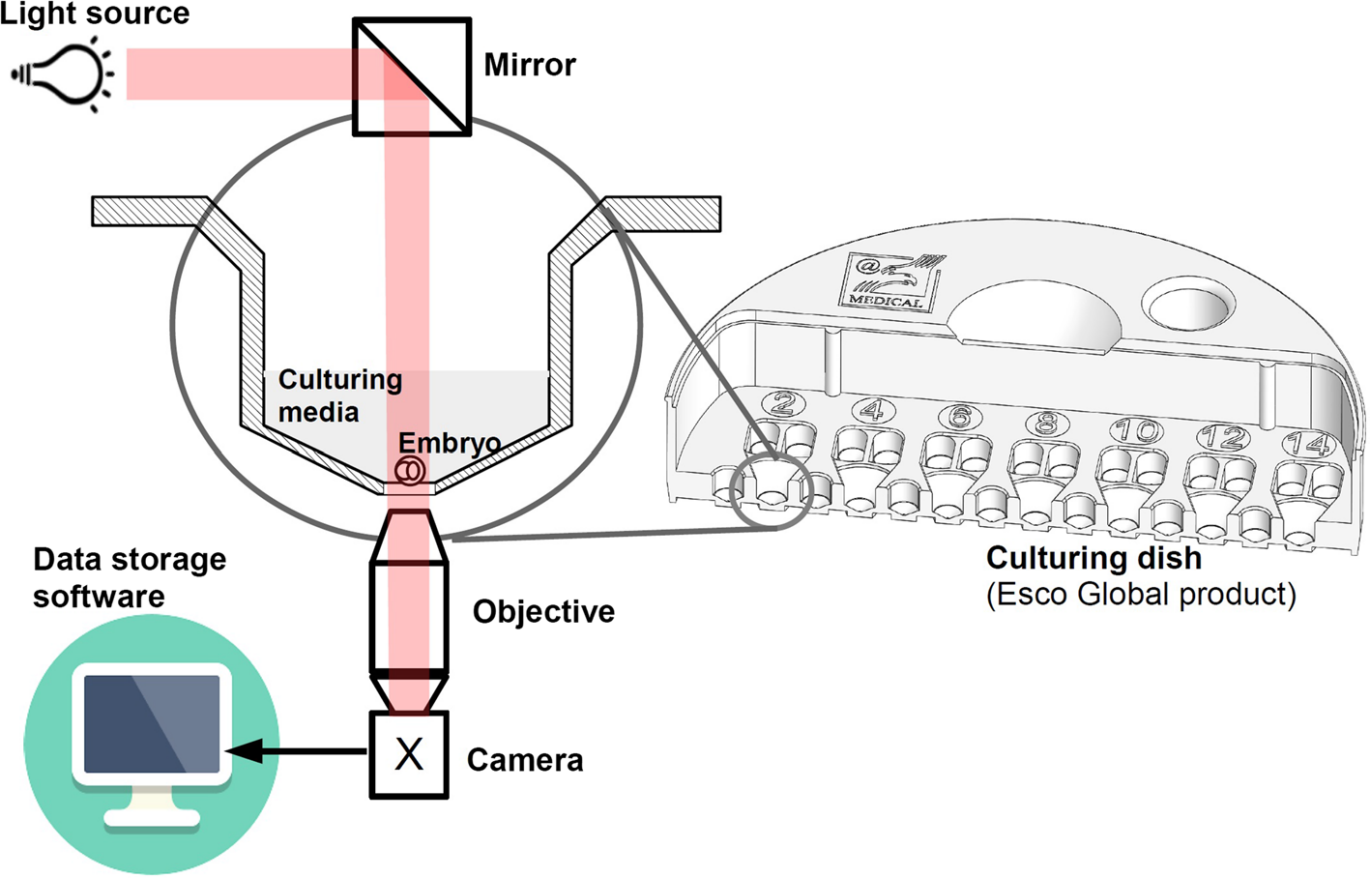
Scheme of time-lapse system

Time-lapse (TL) system is part of the IVF incubator, which is used to register embryo development during its cultivation (see Fig. 6). It captures images of an embryo at certain time intervals (in our case, every 5 min) and stores the images. Typically, such a system consists of three main components: (1) a light source, (2) microscope optics and (3) a video camera. Usually, red light at 650 nm is used to illuminate an embryo, which is cultivated in a specially designed culturing dish, called a culture coin. Microscope optics magnify the embryo cells by 20 times. The TL system is equipped with a 2-megapixels video camera that allows the capture of an embryo in a 121 × 121 μm area. The TL system uses a special mirror (prism) that concentrates light and directs it to the embryo and camera sensor.

Embryo assessment is based on the time intervals between cell cleavages, which are visually registered. The embryo is considered of high quality when the cleavage time intervals fit the normative data. Intervals that are too short or too long between cleavages signal the abnormal development of an embryo, which might lead to pregnancy failure. The TL system facilitates the recording of embryo development for up to 5 days at 5-min intervals to create the sequence of images. Modern time-lapse incubators such as ESCO Miri TL have optical microscopes with which is possible to capture a human embryo at seven different focal planes for more information. Now, embryologists must evaluate each individual image in the sequence and decide which embryo is suitable for transfer. It is a complicated task not only because the embryo can behave unexpectedly during its development, but also because of the massive image data set that includes over 10,000 images per embryo, which must be manually assessed. A skilled embryologist requires less than 2 min to annotate one embryo in the case where embryos do not have a high percentage of fragmentation. Usually, IVF patients have up to 5 or 10 embryos. Henceforth, the manual annotation of all embryos for one patient can take up to 20 min. The automated annotation system can do the same work 10 times faster and without human intervention.

Therefore, an automated detection system of embryo development is presented in the paper that consists of two main components: (1) the localisation of an embryo in an image and (2) the identification of embryo development stages with the aim to identify abnormal division patterns. Since the detection of an embryo localisation in an image is a crucial step, the algorithm is proposed that uses a Haar feature-based cascade classifier to determine the rough embryo location and specify the accurate location with the help of the radiating lines.

### Automatic detection of embryo location

#### Cascade classifier

One of the main steps in this research is to automatically determine embryo location. IVF embryos usually have a round shape with brighter edges. A cascade classifier was trained on a sample containing images with the target object labelled as positives, with negative images containing none of these objects. After the classifier is trained, it can be applied to identify targets in the image. In order to investigate the entire frame, the search window is moved across the image. The search window of a classifier can be easily changed when the size of the target object is unknown. In this case, the search should be performed several times using all possible search window sizes, which are placed on all possible locations in the image [26–28].

**Fig. 7:**
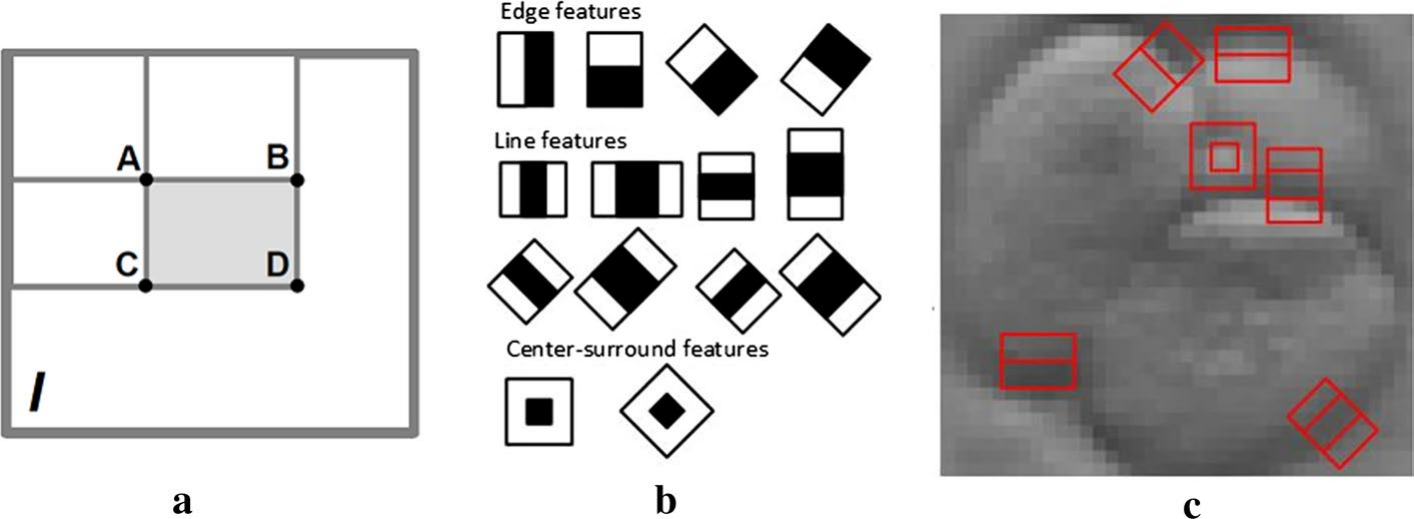
Graphical representation of Haar-like features: **a** simplified example of Haar-like feature represented on integral image; **b** templates of different Haar features; **c** a sub-image of embryo with different feature templates

Cascading is a particular case of ensemble model that is built from several classifiers that are sequentially connected. Learning is a multi-stage process where an extension of the original data by the insertion of new attributes is performed in each step. This process accelerates image processing multiple times, as there is no need to check all of the features that are already learned. Haar-like features (see Fig. 7c) are usually used as inputs to the basic classifiers.

As seen in Fig. 7, Haar-like features are extracted from adjacent rectangular regions at a specific location in a search window. Then, the difference between the sums of the pixel intensities in each region is computed. The numerical value of one Haar-like feature is computed using integral images. The integral images are two-dimensional lookup tables in the form of matrix of the same size as the original image. Each element in the integral image is a sum of all pixels located on the up-left position of the original image. The numerical value or the sum *S* of Haar-like feature is expressed using formula

$$\begin{aligned} S=I(\textit{C})+I(\textit{A})-I(\textit{B})-I(\textit{D}), \end{aligned}$$

where *A*, *B*, *C* and *D* are the points, which belong to the integral image *I*. The sum *S* depends on the type of Haar-like feature to be selected. Usually, a large number of Haar-like features must be retrieved to describe the target object with sufficient accuracy. Therefore, these features are fed into a cascade classifier to construct a strong learner.

#### Proposed algorithm for the detection of embryo location

By default, a cascade classifier allows us quickly to determine the approximate location of an embryo; however, this is not sufficient for solving our problem. Therefore, the embryo location detection algorithm is developed (see Algorithm 1). Embryo detection consists of two main processing steps. The first step involves the application of a cascade classifier for the detection of rough location. A more accurate location of the embryo is then estimated in the next step using the radiating lines over the image filtered by a Sobel filter. Two Sobel operators $G_x$ and $G_y$ are used in this work, which are expressed as

$$\begin{aligned} G_x =\begin{bmatrix} -1&0&1\\ -2&0&2\\ -1&0&1\\ \end{bmatrix}, \quad \\ G_y =\begin{bmatrix} 1&2&1\\ 0&0&0\\ -1&-2&-1\\ \end{bmatrix}, \end{aligned}$$

where $G_x$ is the image gradient in horizontal direction and $G_y$ is the image gradient in vertical direction. Absolute gradient value *G* is given by

$$\begin{aligned} \begin{vmatrix} G \end{vmatrix} = \sqrt{G^2_x+G^2_y}. \end{aligned}$$

The proposed algorithm uses a gray-scale image as an input. The rectangular region of interest (ROI) is returned after the execution of the algorithm. The input image is processed in different scales in order to locate an embryo of the correct size (steps 3–10). If all Haar-like features are applied to satisfy the condition in step 7, then the rough location of the embryo is detected (step 8). A more accurate location (ROI*) of the embryo is estimated in steps 11–15. Sobel filter [29] is used to find the approximate gradient magnitude at each point in the gray-scale image at the ROI (step 11). The radiating lines at each point of the detected square are drawn based on the given parameters, such as line length and the angle between lines. For this purpose, Bresenham’s line-drawing algorithm [30] is applied (step 13). Please refer to Appendix A, for a more detailed explanation of this algorithm. The sum of gradient magnitude for each concentric circle is determined at each point located on the lines. The result of this step is a histogram of obtained values (see Appendix B). The point estimate is computed by determining the maximal value in the histogram and its distance from the centre (step 14). 
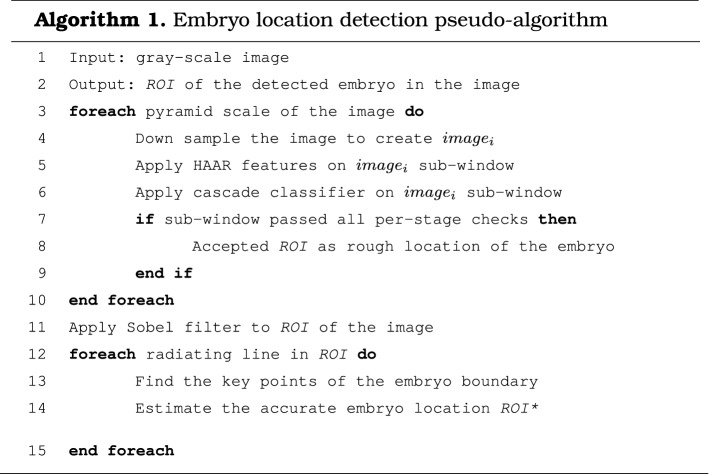


The advantage of the proposed algorithm is the ability to strengthen edges at a substantially equal distance from the central point. Although Sobolev gradient-based optimisers have been used in some previous studies [31–33], the method proposed in this work efficiently uses the traditional optimiser. In addition, the proposed approach is suitable for detecting weak and round curves in a noisy background since it provides successful results without an extra step for noise reduction or intensity normalisation, as seen in previous studies [34, 35]. In comparison, noise reduction is usually applied based on the determined noise types or levels while using traditional methods [36, 37]. For the further processing of images, it is important that the entire embryo is correctly cropped, which is the basis for the determining the cell size, monitoring embryo development stages and then classifying them into defined classes.

Alternatively, this task could be solved using object detection methods such as Local Binary Patterns (LBP) or Histogram of Oriented Gradients (HOG). Both methods were tested, but the cascade classifier was selected for further development. The HOG and LBP methods lack localisation accuracy because they require a high-contrast image, where the target object is captured with sharp edges. Moreover, these methods fail in detecting partially overlapped, noisy or blurred objects, as well as they are too sensitive to object rotation and the location of a region of the target object [38–41]. An embryo image captured using a time-lapse system is slightly blurry and the boundaries of the embryo are too fuzzy; therefore, methods that are able to generalise the results should be employed.

### Identification of embryo development stage by developing a convolutional neural network-based classification system

**Fig. 8:**
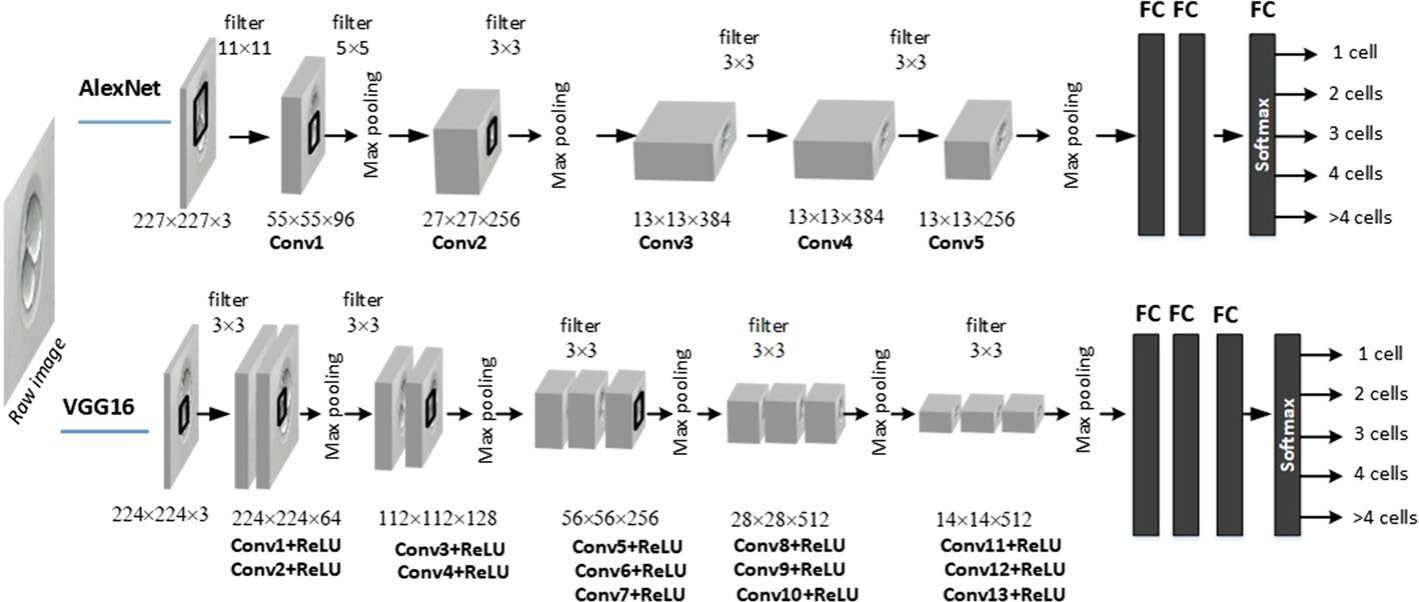
Embryo image classification based on AlexNet and VGG16 architectures

The identification of early-stage embryo development is formulated as a multi-class prediction problem with the aim to identify the cell number during the division process until day 5 of embryo development. The first attempt to solve the given problem incorporated the use of principal component analysis (PCA) and SVM. A cascade classifier was used to detect the location of the embryo in the image. PCA was for the reduction of data dimensionality and feature extraction. SVM was trained to classify different cell stages based on PCA features. The combination of a cascade classifier, PCA and SVM gave approximately 85% classification accuracy. Therefore, we employed CNNs to construct an embryo cell classification system, since CNNs have become one of the most widely used models of deep learning and demonstrate high accuracy performance results in various image recognition tasks [42, 43]. A general CNNs architecture consists of several convolutions, pooling, and fully connected layers. A convolutional layer computes the output of neurons that are connected to the local regions in the input. A pooling layer reduces the spatial size of the representation in order to minimise the number of parameters and computations in the network. These layers are followed by fully connected layers leading to the Softmax layer, which is the final classifier. Two popular architectures, AlexNet and VGG16, were selected for the present experiments (see Fig. 8). Experimental investigations were executed on a Windows 10 machine with 16.0 GB of RAM installed with an Intel Core i7-7700K 4.20GHz CPU. Less than 45 ms were required to process one image and around 1 min (depending on the number of incubating days) was required to analyse entire embryo development from the beginning to end.

AlexNet demonstrates high classification results in different types of applications while retaining a simple and clear structure [44]. As a result, the network of this architecture is easy to implement. The small number of parameters does not require large computational and memory resources. This architecture consists of five convolutional layers and three fully connected layers. AlexNet includes max pooling and makes use of a rectified linear unit (ReLU) nonlinearity which allows training of the network much faster compared to using a common activation function (e.g. tanh or sigmoid) together with data augmentation and dropout regularisation in order to avoid overfitting.

VGG16 network [45] is an improvement over AlexNet by providing the deeper architecture. A total of 16 layers exist in this architecture, including 13 convolutional layers and 3 fully connected (FC) layers followed by a Softmax classifier. In VGG16, large kernel-sized filters in the first convolutional layers ($11\times 11$, $5 \times 5$) are replaced with multiple $3 \times 3$ filters that are used in all 13 convolutional layers. Max pooling layers use only a $2 \times 2$ px window with stride of 2. For all convolutional layers, the stride and padding are set to 1 px.

Comparison of these two architectures reveals that VGG16 has twice as many parameters ($\sim$527 MB of required memory) as AlexNet ($\sim$232 MB of required memory), which makes it more likely to observe VGG16 demonstrating $\sim$15% higher classification accuracy over AlexNet [46]. However, the computational complexity of VGG16 is very high, being 10 times greater than that of AlexNet. Notably, AlexNet is one of a few CNNs models capable of achieving super real-time performance with very small batch sizes, thus allowing it to reduce the consumption of system memory (e.g. a batch size of 1 requires less than 1 GB memory). In this research, both architectures are used to explore and estimate their possibilities of achieving high accuracy results (more than 90%) in identifying a total cell number in images of an embryo.

The classification model has been implemented using MatConvNet [47], an open-source implementation of CNNs in the MATLAB environment that can be easily extended in order to develop new CNNs architectures. Specific software and hardware requirements exist for deep learning model implementations, such as MATLAB 2016a (or later version), a C\C++ compiler, and a computer with a CUDA-enabled NVIDIA GPU supporting compute capability 2.0 or above.

In general, different types of measures are used to evaluate the performance of the selected classifiers. In the multi-class setting, the outcome is produced for many predefined classes $\{C_1, \ldots , C_i, \ldots ,C_K\}$, where *K* is the class cardinality [20, 21]. Accordingly, for an individual class $C_i$, the main counts are defined as true positives $TP_i$, false positives $FP_i$, false negatives $FN_i$, and true negatives $TN_i$. These are the main entrances for the confusion matrix. A list of measures used to assess the performance of a multi-class predictor is richer compared to binary classification. The conventional performance measures are modified to consider the class distribution resulting in macro-averaging or micro-averaging computation. A macro-average defines the performance treating all classes equally, whereas a micro-average considers the contributions of all classes to compute the selected measure. Obviously, in a multi-class setting, a micro-average is preferable if the class imbalance is prominent.

## Figures and Tables

**Fig. 9:**
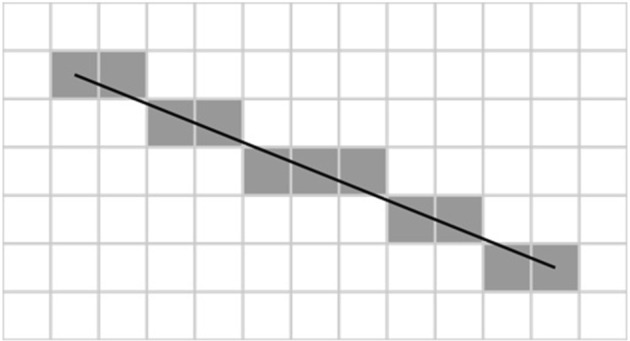
Example of a straight line drawn using Bresenham’s algorithm

**Fig. 10:**
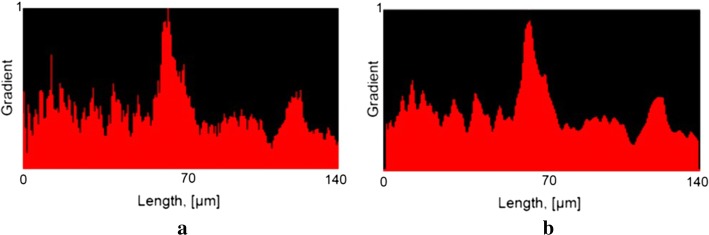
Example of aggregated histogram of gradients (**a**) and filtered histogram of gradients (**b**)

## Data Availability

The image data set used to support the findings of this study has not been made publicly available because the images are owned by a private IVF laboratory (ESCO MEDICAL Ltd., company code 303705851, Draugystes str. 19, 51230 Kaunas, Lithuania) and are available by request only.

## Appendix A

### Bresenham’s line-drawing algorithm

The line-drawing algorithm determines the pixels on 2D digital image that should be selected in order to get close approximation of a straight line between two points. Line example is shown in Fig. 9.

The algorithm is based on incremental error computation. There exist two basic assumptions concerning the particular implementation of the algorithm. First, the beginning of coordinates is the top-left corner. The pixel coordinates increases in right and down directions. Second, the pixel centres have integer coordinates. Line-drawing algorithm selects the integer *y* coordinate corresponding to the pixel, which is the closest to the ideal *y* coordinate. General line equation is given by:

$$\begin{aligned} \frac{y - y_0}{y_1 - y_0} = \frac{x - x_0}{x_1 - x_0}, \end{aligned}$$

where $x_0$, $y_0$ are the beginning coordinates of the straight line and $x_1$, $y_1$ are the end coordinates. The final value of coordinate *y* is estimated by rounding the quantity to the nearest integer value. To clarify, the pseudo-code of the Bresenham’s line-drawing algorithm is given in Algorithm 2.

## Appendix B

The histogram of gradients is estimated by aggregating the gradient values computed for each radiating line. The distance from the centre of the embryo to the end of the radiating line is shown on the horizontal axis in $\upmu$m. The aggregated and normalised gradient values are shown on the vertical axis (Fig. 10).

Filtered version of aggregated histogram of gradients is used in this work. The embryo boundaries (or edge) are determined based on the given histogram. The highest gradient values are acquired in the image areas where rapid changes in colour intensities appear. These regions are usually the boundaries of the embryo cell. The distance from the beginning of the histogram to its peak determines what radius for embryo localisation should be selected in the analysis. The filtered histogram is shown in Fig. 10b).

## Appendix C

See Table 4.

Results of tenfold stratified cross-validation.

**Table 4:** Average classification accuracy

Fold number	VGG architecture					Avg	AlexNet architecture					Avg
	Embryo development stages						Embryo development stages					
	1	2	3	4	> 4		1	2	3	4	> 4	
1	0.928	0.924	0.904	0.939	0.923	0.923	0.910	0.927	0.903	0.913	0.911	0.912
2	0.940	0.956	0.947	0.979	0.938	0.952	0.924	0.951	0.944	0.949	0.916	0.936
3	0.933	0.927	0.925	0.950	0.931	0.933	0.912	0.947	0.915	0.921	0.911	0.921
4	0.951	0.961	0.955	0.958	0.925	0.950	0.933	0.935	0.932	0.920	0.937	0.931
5	0.943	0.919	0.912	0.914	0.902	0.918	0.916	0.938	0.899	0.937	0.911	0.920
6	0.949	0.947	0.910	0.944	0.927	0.935	0.929	0.931	0.921	0.934	0.938	0.930
7	0.929	0.932	0.921	0.934	0.914	0.926	0.931	0.962	0.939	0.928	0.917	0.935
8	0.935	0.958	0.952	0.959	0.933	0.947	0.921	0.921	0.930	0.956	0.932	0.932
9	0.945	0.932	0.913	0.957	0.907	0.931	0.926	0.935	0.908	0.933	0.902	0.920
10	0.926	0.956	0.953	0.968	0.924	0.945	0.918	0.953	0.909	0.937	0.944	0.932
Average	0.938	0.941	0.929	0.950	0.922	0.936	0.922	0.940	0.920	0.933	0.922	0.927
St. deviation	0.009	0.016	0.021	0.018	0.011	0.012	0.008	0.013	0.016	0.013	0.014	0.008

## Abbreviations

CNNs: convolutional neural networks
FC: fully connected
IVF: in vitro fertilisation
PCA: principal component analysis
ReLU: rectified linear unit
ROI: rectangular region of interest
SVM: support vector machine
TL: time-lapse
